# Keyword Index for Volume 99

**DOI:** 10.1038/sj.bjc.6604832

**Published:** 2008-12-09

1q 1136

^131^I 632

[^18^F]FDG 481

[^18^F]FLT 481,

2-deoxy-d-glucose 1842

2-methoxyoestradiol 1433

2-methoxyoestradiol-3,17-*O*,*O*-*bis*-sulphamate 1842

4T1 model 741

5-fluorouracil 716, 1020

7p 774

8q 774

*α*-fetoprotein 72

ABC transporter 1651

access to health care 1040

accrual rates 1967

*ACSL3* 314

*ACSL3: ETV1* fusion 314

active immunotherapy 350

adenovirus 1668

adherence 428, 1763

adiponectin 1246

adjuvant chemotherapy 597, 1226, 1232

adjuvant treatment 875

adolescent HPV vaccination 1908

adrenocortical carcinomas 1900

adult gliomas 1153

advanced breast cancer 1984

advanced cancer 1251

advanced gastric cancer 858

advanced solid tumours 1387

aetiology 545

AFP 72

age 1007, 1046

age at menarche 201

age inequalities in cancer care 1967

AIDS 800

AKT 1265, 1296

AKT1 488

albumin 1013

alcohol 805

allele-specific PCR 90

anaemia 14

anaplastic 781

androgen receptor 1743

angiogenesis 160, 321, 622, 1348, 1375, 1387, 1433, 1557, 1961, 2054

angiotensin receptors 160

antagonists and inhibitors 616

anthropometric factors 207

anthropometry 201

antiangiogenic 1197

anti-angiogenesis 734, 1204

antibody-drug conjugate 100

anti-cancer drug 1442, 2044

anti-CEA antibody targeting 632

anti-EGFR therapy 868

antiretroviral drugs 800

anxiety 1794

AP-1 1269, 2013

APC 1735

Apo-2L 294

apoptosis 283, 294, 841, 989, 1442, 1816, 1823

ARE1 -158 G/A polymorphism (rs266882) 1743

aromatase inhibitor 428

AS1404 2006

ASA404 2006

asbestos 173, 822

asbestos removal 822

astrocytomas 160, 294

atypical squamous cells of undetermined significance (ASCUS) 563

automated fluorescence microscope 789

awareness 1593

azathioprine 1276

*β*2-microglobulin 1453

basal cell carcinoma 1276

Bayesian analysis 1786

BCL-2 family proteins 1613

behavioural intention 225

bevacizumab 93, 1564

biliary tract cancer 811

biliary tract carcinoma 862

bioluminescent imaging 2037

biomarkers 655, 841, 1103

birth weight 1161, 1544, 1916

*bis-*sulphamates 1433

biweekly schedule 455

BK virus 1383

bladder cancer 1383

BLT2 1064

BMI 1517, 1912

*BNIP3* 727

body mass index 185, 527, 811, 1916

body size 196, 1517

*BRAF* 83, 2020, 2065

*BRAF* gene 1673

brain tumour 796

brain tumour stem cells 2044

*BRCA1* 371, 974

BRCA1/2 1302, 1534

*BRCA2* 371, 974

breast 327, 1842

breast cancer 68, 196, 335, 387, 404, 408, 415, 423, 428, 513, 539, 597, 604, 704, 711, 774, 930, 974, 1000, 1089, 1170, 1185, 1221, 1226, 1357, 1369, 1433, 1502, 1534, 1544, 1623, 1644, 1763, 1775, 1940

breast cancer cell line 1246

breast cancer cells 481

breast cancer metastases 741

breast cancer risk 201, 207

breast cancer screening 1176

breast carcinoma 63, 611, 639

breast neoplasms 616

breast volume 1534

cachexia 2029

calcium 1539

cancer awareness 1221

cancer incidence 191

cancer registries 1923

cancer screening 655

cancer stem cells 387

cancer stromal cell 305

cancer survival 830, 1923

cancer susceptibility 670

cancer-associated fibroblasts 1375

capecitabine 584, 591, 858, 1316

carbonic anhydrase 9 1468

carboplatin 51, 894

carcinogenesis 1961, 2070

carcinoma 245, 1493

cardia 1506

case–control study 423

caspases 283

caveolin 1 327, 1684

caveolin 2 327

c-Cbl 1849

CCI-779 1197

CD133 100, 1285

*CDKN2A* 364

cDNA microarray 335

C/EBP*β* 781

cell cycle 1635, 1823

cell invasion 1114

cell lines 1330

cell signalling 750

central obesity 527

Cervarix 1908

cervical cancer 225, 1096, 1216, 1557, 1651, 1929

cervical intraepithelial neoplasia 239

cervical intraepithelial neoplasia (CIN) 563

cetuximab 83, 455

c-Fos 1269

CFU_gm_ 632

CGH 1136

ChA 72

chemoendocrine therapy 1564

chemokines 930, 1493

chemo-radiation 57

chemoradiotherapy 1020

chemosensitising 1832

chemosensitivity testing 760

chemotherapy 6, 23, 44, 51, 57, 63, 266, 283, 442, 481, 591, 695, 868, 900, 1000, 1395

chemotherapy response 387

chest X-ray 557

childhood 796, 1129, 1529, 1539, 1916

childhood cancer 179

childhood leukaemia 219, 1668

childhood nutrition 1517

China 196, 811

CHK2 or CHEK2 (checkpoint kinase 2) 1302

choroid 1673

chromosome damage 670

chronic myelogenous leukaemia 387

CI-1040 2020

ciliary body 1673

circulating tumour cells 789, 841

cisplatin 167, 442, 852

c-KIT 2065

claudin-4 491

clear cell carcinoma 1197

clinical trials 1593, 1967

coffee 1534

cohort study 191, 1165, 1946

collagen IV 68

colon cancer 966, 2094

colorectal 275, 1285

colorectal cancer 133, 136, 266, 459, 532, 632, 716, 727, 1046, 1050, 1239, 1316, 1511, 1607, 1718, 1729, 1867, 1923, 1991, 2088

colorectal, prostate and ovarian cancer 789

combination 1

combination therapy 689

combined modality therapy 900

combretastatin 321

common gene activation 1307

community 1763

compliance 428, 1593

concurrent chemoradiation 167

contralateral cancer 539

corticosteroids 23

cost-effectiveness 230, 557, 1984

Cox regression 179

CpG island 136

cribriform patterns in thyroid tumour 1874

c-Src activation 1769

CT screening 557

curative surgery 1046

cure 219

CXCL13 930

CXCR4 1623, 1695

cyclooxygenase-2 1729

CYP1B1 1056

CYP2C19 1251

CYP3A4 1239

CYPIA2 1534

Cyr61 1656

cystine transporter 464

cytochrome P-450 2D6 616

cytogenetic 1673

cytokeratins 841

cytotoxic agents 1

dasatinib 1074

deep vein thrombosis 1034

definitive chemoradiotherapy 1586

depression 1794

depressive mood 1408

deprivation 1923

dermcidin 126

dexamethasone 2054

diabetes 1380

diagnostic marker 939

dietary folate 816

dietary patterns 191, 1511

differential expression 1136

differentiation 1849

disease progression 1572

disease-free survival 966

disease-specific survival 539, 1476

distant metastases 513

distress 1975

diuretics 1522

DKK3 663

DLEC1 375

Dll4 1204

DMXAA 2006

DNA methylation 136

DNA microarray 1484, 1635

DNA-repair genes 1050

docetaxel 852, 1426, 1832, 2054

dose 275, 722

dose fractionation 632

dose intensity 253, 1226

dose reduction 259

doxorubicin 442, 1816

drug metabolism 1251

drug resistance 118, 387, 464, 473, 1808

drug targeting 392, 900, 1256

Du145 1656

dynamic MRI 321

early life 796, 1544

eastern Europe 1912

E-cadherin 1832

economic evaluation 1232

economics 1991

ECX 868

efficacy 584

efficiency 2001

EGCG 647, 1056

EGFR 83, 245, 341, 911, 923, 1056

EGFR inhibitors 473

EGFR-targeting drugs 1

EGFRvIII 341

elderly patients 51, 584, 1586

elective 1046

EndMT 1375

endocrine-responsive breast cancer 1564

endometrial adenocarcinoma 1034

endometrial cancer 434, 1651

endometrial stromal sarcoma 1210

endosialin 1348

endothelial 1204

endothelial-to-mesenchymal transition 1375

Endothelin-converting enzyme-1 (ECE-1) 1114

endothelium 1256, 1375

engineered antibody 1415

enzastaurin 750

ependymoma 1129, 1136

EphA2 1074

EPIC Study 191

epidemiology 1522, 1529, 1946

epidermal growth factor receptor 90, 1729, 1757

epigenetics 136

epirubicin 858

epithelial 1476

epithelial cell adhesion molecule (EpCAM) 1635

epithelial–mesenchymal interactions 151

epoetin-*β* 14

Epstein–Barr virus 1816

ErbB 1415

ERCC1 167

ERG 847

ERK1/2 2020

erlotinib 93, 911

ER-positive breast cancer 1769

ethnicity 1040, 1757

ETS 847

Ets-1 1348

*ETV1* 314

EXE 858

exercise 30

expert rating scale 37

exploratory trial 415

faecal tumour M2 pyruvate kinase 133

false-positive cases 239

familial predisposition 1748

familial risk 536

family history of breast cancer 1007

Fas ligand 502, 1600

Faslodex 1984

fatigue 23, 30, 1408

*FBLN1* 2083

female neoplasms 816

fibroblast growth factor receptor 2, 10, 305

fibrosis 1375

focus genes 1307

FOLFOX 1395

follow-up 704

Frizzled 143

FTIR microspectroscopy 1859

fuel 1934

fulvestrant 1984

fusion 847

G3 endometrioid endometrial carcinoma 768

GA733 1290

gallstones 811

gastric 100

gastric adenocarcinoma 949

gastric cancer 126, 584, 1307, 1322, 1802, 2083

gastrointestinal stromal tumour 448, 1600

gemcitabine 760, 862, 1387

GEMOX 862

gender 1506

gene expression 1330

gene expression profiling 1307, 1729, 1884

gene regulation 978, 1246

gene signature 1884

genetic susceptibility 536, 1748

genome profiling 387

genomic instability 1121

genotyping 563

germ cell tumour 1748

*γ*H2AX 1129

Gleason grade 1859

glioblastoma 294, 1678, 2044

glioma 185, 294, 1144

glomerular filtration rate 894

glucose level 1380

glutathione 464

glycaemic index 434, 1170

glycaemic load 434, 1170

glycolysis 989

GOLPH2 939

GP73 939

granulocyte colony-stimulating factor 253

Great Britain 822

Greece 191

green tea 1179

green tea polyphenol 647

group rehabilitation 1975

GSTP1 1316

GTG banding (G-bands after trypsin and giemsa) 1302

Guthrie card 1668

*H19* 1891

hCG*β* 72

HDAC inhibitors 1623

head and neck cancer 57, 93, 167

health-related quality of life 1975

height 185, 1916

hepatitis C 805

hepatoblastoma 1891

hepatocarcinogenesis 151

hepatocellular 100

hepatocellular carcinoma 143, 805

hepatocyte growth factor 1623

HER family 1775

HER-2 68, 520

HER-2/neu 341

hereditary non-polyposis colorectal cancer 1726

heritable tumour syndromes 683

heterogeneity 1673

HIF-1*α* 459, 1442

HIF-2 1348

high-risk HPV 404

highly sensitive *in situ* hybridisation 350

histone deacetylase 689

HIV 800

Hiwi 1083

HL60 670

HLA class I antigen 1462

HNF3*β*/FoxA2 781

HNSCC 357

home 1934

hormonal therapy 611

hormone receptor 196

hormone receptor positive 1984

hormone refractory 1296

hormones 1161

HPV 214, 230

HPV-16 408

HPV prevalence 1929

human chorionic gonadotrophin subunit *β* 72

human papilloma virus (HPV) 225, 408, 563

human tissue kallikreins 1103

hypermethylation 357

hypertension 1912

hypothyroidism 448

hypoxia 727, 1348, 1442, 1468

hysterectomy 1216

Id-1 1557, 1832

Id-1 gene 404

IGF binding protein 3 1096

*IGF1R* 83

*IGF2* 1891

IGFs 1357

imatinib 734, 1600

immune evasion 1867

immune function 2029

immunohistochemistry 321, 327, 335, 639, 939, 957, 1285, 1453, 1704, 1712

immunosuppression 1276

implementation 415

*in utero* 545

incidence 176, 182, 1502, 1940

Indonesia 214

induction 57

infection 1529

inflammation 1251, 2029, 2070

inflammatory breast cancer 1735

inflammatory cells 1013

information 1908

informed 1007

infrared spectroscopy 1859

inhibitor 689, 911

inositol hexakisphosphate 1613

insulin-like growth factor-1 receptor 1096

integrative approach 1307

integrin *β*1 1684

intercostobrachial neuropathy 604

intra-uterine growth 179

intraperitoneal therapy 2037

invasion 663, 1322, 1453

involution 1369

ionising radiation 1940

IPMN 1064

ipsilateral breast relapse 539

IR (ionising radiation) 1302

irinotecan 275, 455, 577, 1239, 1316, 1607

irradiation 93

isothiocyanates 1823

Italy 173, 225

Jak-Stat pathway 2044

Japan 176, 1502, 1179

Kaposi sarcoma 800

Kdr 1204

Ki-67 1013, 1121

Ki-67/MIB-1 72

kidney cancer 1912

KIT 2065

*KLHL9* 364

*KLK6* 1484

*KLK10* 1484

knowledge 1007

*KRAS* 83, 245, 923, 2020

kRT-PCR 1775

lapatinib 711

laser microdissection 1735

laser-capture microdissection 1849

lentivirus 622

leukaemia 1529

leukotriene B_4_ 1064

life expectancy 1991

liposomes 392, 1256

liquid-based cytology (LBC) 563

*Listeria monocytogenes* 741

LIV-1 774

liver metastasis 350, 716, 957, 1729

LKB1 245, 683

localised 1027

locoregional recurrence 1121

long-term effects 30

longitudinal study 1794

loss of heterozygosity 1891

loss of imprinting 1891

low-dose rate exposure 1940

low-grade squamous intraepithelial lesion (LSIL) 563

LPA 1322

lung cancer 173, 557, 683, 852, 911, 1179, 1635, 1757, 1934

LXXLL motif 639

LY294002 2013

lymphangiogenesis 110

lymphoid leukaemia 488

lymphoma 1387

MAGE-A protein 350

MAGE-A10 mRNA 350

Mage-b DNA vaccine 741

MAL 1802

malignancy 545

malignant neoplasms 1549

malignant pleural mesothelioma 44, 51

mammary epithelial cell 1246

mammographic density 1369, 1539

mammography 1007, 1176

mammography screening 1185

MAPK 1089, 1823

margins 357

marker expression 491

mass screening 239

maternal age 1161

MDA-MB-231 1056

*MDM2* 1144

Mdm2 SNP309 78

Mediterranean diet 191

MegaFasL 1600

melanoma 536, 1265, 1673, 1678, 2065

melanoma-prone families 364

meningioma 182, 185

menopausal status 196

*MET* 83, 911

meta-analysis 434, 1170, 1340

metabolism 989

metastasis 110, 398, 923, 957, 1718

metastatic breast cancer 1572

metastatic colorectal cancer 455, 577, 722

metastatic gastric cancer 1402

metastatic melanoma 734

methionine 816

methylation 1735, 2083

methylation-specific PCR 1735

microarrays 398, 768, 1330, 1729, 1884

microenvironment 1484

microsatellite instability 1607, 1726, 1867

microtubule 1842

microvessel density 321, 1013, 1961

middle aged 239

midkine 655

MI-ER1 639

migration 1322, 1453, 1493

mismatch repair 375

mitochondria 989, 2088

mitomycin C 591

MnSOD 283

modelling 1991

molecular diagnosis 1859

molecular markers 2001

mortality 173, 253, 822, 1549, 1763, 1934

motesanib diphosphate 1387

motility 520

*MRE11* 1607

mRNA 1775

MSC 622

MT1-MMP 663

mTOR 1056

mTORC2 1197

MUC1 978

MUC4 520, 949

mucin 949

mucosal 2065

multiparametric analysis 1103

multiple primary neoplasm 1340

multiplex ELISA 841

multiplex ligation-dependent probe amplification 364

muscle strength 30

mutation screening 90

mutations 245, 488, 923, 1265, 2020

*MYCN* gene 1027

nanomedicines 392, 900

National audit 695

national cancer statistics 830

natural killer-cell malignancies 1816

NB7M 1823

NEAT 1226

neoadjuvant 1020

neoplasm 1408, 1506

neprilysin 1114

NET1 1322

network mapping 1307

neuroblastoma 1027, 1165

neuroendocrine markers 1330

neuroendocrine tumours 72, 1330

neuropathic pain 604

neuropilin 1153

Nfkb1 2070

niacin 816

NLS (nuclear localisation signal) 1302

non-clear cell histology 259

non-Hodgkin's lymphoma 253

non-seminoma 1517

non-small cell lung cancer 245, 750, 852, 2006, 2013

normal mucosa 136

Norway 1165

Notch1 1204

Nrf2 2070

NSCLC 375, 923, 1476

nuclear 1769

nuclear factor-*κ* B 1816

nuclear localisation 639

nucleoside transporter 760

nucleosomal DNA 841

nutrition 2029

obesity 527, 811, 995

occupational exposure 822

oesophageal cancer 126, 1020, 1395, 1586

oesophageal squamous cell carcinoma 1462, 1468

oesophagogastric cancer 868

oesophagus 1506

oestrogen receptor 639, 774

older women 1221

oral cancer 1121

oral cancer invasion 647

oral contraceptive 1534

oral squamous cell carcinoma 655, 1453

organ transplant recipients 1276

orthotopic 93, 110

outcome 727

ovarian cancer 283, 341, 434, 520, 1103, 1269, 1794, 1823, 1916, 2020, 2037

overdiagnosis 1176

oxaliplatin 577, 858, 862, 1020, 1050, 1395

p14^ARF^ 364, 1144

p16^INK4a^ 110, 364

P38 1808

p53 118, 974

p53 pathway 78

paclitaxel 1678, 2037

paediatric oncology patient 23

paediatrics 894

pancreas 622

pancreatic cancer 6, 110, 126, 305, 464, 527, 760, 1064, 1074, 1083, 1290, 1484

pancreatic cancer progression 1695

PanIN 1064

papillary 781

papillary thyroid carcinoma 1874

par6 1476

parents 1908

parity 1161

patient values 875

patient–doctor agreement 415

PCa 1656

PC-DFA 1859

pemetrexed 51, 750

perception 1593

perinatal 1165, 1544

PET 481

Peutz-Jeghers syndrome 683

pharmacodynamics 1579

pharmacogenetics 716, 1050, 1251

pharmacogenomic 1757

pharmacokinetics 1239, 1387, 1433, 1579

pharmacology and therapeutic use 616

pharmacovigilance 253

phase I 1579

phase II 734

phosphatase 1718

phosphatidylinositol 3-kinase pathway 2013

photosensitising reaction 1522

physical activity 1408

physical status 408

Physicians’ Health Study 1743

PKC*α* 1808

PKC*δ* 1808

plasma D-dimer 1034

platelet-derived growth factor receptor 1426

pleural cancer 173

ploidy 513, 1121

PMPS 604

polymer therapeutics 900

polymers 392

polymorphisms 716, 978, 1050, 1357

population based 214, 1506

positivity criterion 415

positron emission tomography 989

postnatal growth 1544

postoperative pain 604

potassium channels 989

PPAR*γ* 2044

PRAME 398

prediction of therapy 1103

predictions 1549

predictive value 1775

pre-malignant 1961

prenatal infection 1668

preoperative therapy 852, 1564

prevalence 214

prevention 532, 995

primary breast cancer 1013

primary chemotherapy 68

primary culture 1678

primary tumour 923

principal components analysis 1511

PRL-3 1718

PRMT1 2094

procoagulants 1000

prognosis 160, 335, 341, 357, 375, 398, 539, 655, 1083, 1089, 1129, 1285, 1462, 1468, 1651, 1712, 2094

prognostic factors 1027, 1210, 1269, 1402

prognostic indicator 1557

prognostic marker 1802, 1884

prognostic value 1775

progression 1269

proliferation 335, 1635, 1635, 1884

proliferation signature 966

proliferative activity 513

prominin-1 100

promoter hypermethylation 1802

promoter methylation 375

promoters 1144

prophylactic HPV vaccine ‘CervarixÔ’ 1929

prospective cohort studies 176, 1179, 1502, 1511

prospective study 448

prostaglandin E_2_ 502

prostate 1296, 1842

prostate cancer 78, 126, 176, 314, 371, 491, 847, 939, 1040, 1114, 1426, 1613, 1743, 1786, 1832, 1849, 1859, 2054

proteasome inhibitors 1613

protein arginine methyltransferase 2094

protein kinase C*β* 750

protein kinase C*δ* 1644

protein tyrosine kinases 734

PSA gene 1743

pseudomyxoma peritonei 591

psycho-oncology 37

psychosocial distress 37

psychosocial support 1975

PTCH gene 1276

PTEN 341, 1089, 1296

PTK6 (BRK) 1089

pulmonary 597

pulmonary thromboembolism 1034

QMSP 357

Q-TWiST 711

quality of life 30, 415, 1226

quality-adjusted survival 711

Rac1 1656

*RAD50* 1607

radiation 545, 1216, 1946

radiation sensitivity 670

radioimmunotherapy 632

radiology 1185

radiosensitisation 1442

radiotherapy 63, 266, 900, 1395

radon 1946

randomised clinical trial 442

rapamycin 1197

receiver operating characteristic curve 1712

receptor tyrosine kinase 1074

*RECK* hypermethylation 647

record-linkage study 805

rectal cancer 1232, 1712, 1923

recurrence 357

registries 182

regulation 502

regulatory T cells 1867

rehabilitation 30

relative survival 1786

renal cell cancer 259, 448, 1380

renal function 894

resection 266

residual disease 1216

resistance 911

response 275, 1020

RET/PTC3 and E7 transgenic mice 1874

review 683

RFLP 78

RhoA 1322

riboflavin 816

risk 1369, 1522, 2088

risk factors 179, 545

risk modelling 557

RNA and protein 491

RNAi 1695

S-1 584

S100 proteins 1136

safety 584

SAGE 1136

salivary adenoid cystic carcinoma 90

salivary glands 90

Sam68 1089

sandwich ELISA 768

schedule dependency 1808

schools 1908

screening 133, 532, 557

second cancers 253, 536, 611

second-line chemotherapy 1402

securin 335

semaphorin 1153

seminoma 1517

serine proteases 1103

serotonin reuptake inhibitors 616

serum collagen IV 68

serum markers 768, 930

service screening 423

sex steroids 201

shared decision-making 875

signalling pathways 1623

signet ring cell carcinoma 949

Singapore Chinese 1511

single and multiple HR cases 1929

single nucleotide polymorphism 1340

size at birth 201

skin cancer 1522

sleep duration 176, 1502

sleeping problems 1408

Smad7 957

small-cell lung cancer 442

smoking 1161, 1912

smooth-muscle myosin 1726

Snail 1900

SNP309 1144

socioeconomic deprivation 830

soy 196

spatial models 1786

splicing 978

squalene epoxidase 774

squamous cell carcinoma 167

squamous cell carcinoma of the head and neck 1684

Src 1684

stage IB/II 852

stage-III 1718

STK11 683

stomach cancer 350, 1704

stool testing 133

stopping rule 2001

stove 1934

stromal 1476

SULT1A1 1340

sunitinib 259, 448, 1380

suppressor 1114

surgery 63

survival 14, 63, 219, 266, 398, 1013, 1046, 1210, 1269, 1285, 1316, 1357, 1426, 1572, 1900, 2001

survival analysis 1704

sustained chemotherapy 2037

Swiss HIV cohort study 800

systemic therapy 695

TAM67 2013

tamoxifen 428, 616, 1056, 1763, 1769

targeted therapy 473, 1290

TEAD1 1849

Techa River 1940

teenagers and young adults 830, 1967

tegafur–uracil 577

telatinib 1579

telephone follow-up 704

telomerase 1129

testicular cancer 1517, 1748

testicular microlithiasis 1748

TGF-*β*1 957, 1357

thiamin 816

thymidine kinase 481

thymidylate synthase 716

thyroid cancer 781

tissue array analysis 1704

tissue microdissection 1083

TMPRSS2 847

tobacco related cancer 1340

tocotrienol 1832

topoisomerase II*á* 670

toxicity 259, 275, 597, 1316

*TP53* 727, 1144

T_pot_ 1678

transcript 978

transcription 663

translocation 847

transplant recipients 1383

treatment guidelines 875

treatment preferences 875

trefoil factor 768

trends 219, 830, 1549

trichostatin A 1623

TROP2 1290

tumour advancement 1557

tumour antigen 398

tumour expression 930

tumour growth 1493

tumour hypoxia 459

tumour immune evasion 502

tumour immunity 1867

tumour marker 72, 1712

tumour microenvironment 118

tumour proliferation 2037

tumour stroma 118, 151

tumour suppressor gene 2083

tumour-infiltrating lymphocyte 1704

tumour-VDA 2006

tyrosine kinase inhibitor 1380, 1579

UFT 577, 722

UGT1A1*28 275

UGT1A1 gene polymorphism 1239

ultrasound 1748

U-miners 1946

upper aerodigestive tract cancer 1340

uracil–tegafur 1232

urban–rural 207

urothelial cell carcinoma 663

uterine cancer 611

vaccination 230

vaginal smears 239

vascular disrupting agents 1256

vascular endothelial growth factor receptor 1579

vascular maturity 321

VDA 2006

VEGF 622, 1153, 1380

venous thromboembolism 1000

vimentin 1476

vinorelbine 44

viral aetiology 1668

viral load 408

vitamin D 1539

waist-to-hip ratio 527, 811

waiting times 695

weight gain 995

weight loss 995

WNT 143, 1695

women 185

xCT 464

xenografts 1074

X-irradiation 1442

